# The Prolongation of Life

**Published:** 1913-10

**Authors:** 


### Abstracts and Selections.

### The Prolongation of Life.

Up to within recent times the search for the longer life has been merely a groping in the dark, and following no definite course. It was largely a search by individuals from most selfish motives and without even an attempt at concert, and it remained for the science of the advanced modern type to place this quest in the category of the material, to be sure, but of value to the community at large as well as to the individual. Civilization has for its greatest achievement, and almost entirely through the medical sciences, the conservation and the prolongation of life. The goal is to breed a race of longest life, of most efficiency, and with the least possible waste. During the ages when constant warfare and its mate, the ever present epidemic, wiped out whole peoples, life had no value other than as a subject for destruction. Then the average span of life was about twelve years. But with the dawn of enlightenment, promiscuous warfare is gradually ceasing, and the advances in the science of life have progressed so rapidly that we have been able to reach the present high average length of life.

And now, even after these adverse conditions have been overcome, the death rate is yet too high. A high infant mortality still confronts us, though even here there is a large improvement upon previous conditions. Typhoid fever, cancer, and the degenerative diseases form a very large proportion of the death rate. In recent years, however, typhoid fever has been reduced about forty per cent. On the other hand, cancer has risen over 100 per cent, and the public and the profession are now fairly alive to the danger, and are enthusiastically taking up the campaign of education along the lines of prevention.

Industrial diseases form a very large drain on life. In recent years the growth of the industries has been so rapid that the development of safety provisions is unable to keep pace with them. As fast as possible, however, remedial measures are shaping themselves and results are already manifesting themselves in the remarkable decrease in the death and disease incidence in these industries. The health authorities have taken hold, and they have made industrial disease reportable, and as a result legislative enactments are meeting the varying demands as they arise.

Child labor is receiving its quota of attention. Its deleterious influence on the child's health and length of life is under scientific investigation. Many States have already enacted prohibitive legislation, and only within a short time a most radical suggestion has been offered by one of our law makers—Senator Beveridge of Indiana—namely, to have the United States prohibit the transportation of all goods made by child labor in interstate and foreign commerce, on the ground that such articles are not "legitimate articles of commerce"—and thus to wipe out this practice entirely. To offset this, however, David Heron found that in the Yorkshire, England, woolen mills before legislation prohibiting child labor was enacted, the families in that section were large and well kept, because children were assets, while now the size of the family is kept down, and the children neglected, because the mothers are compelled to take their places in the factories. Investigations conducted along the same lines in our Southern States, where child labor is common, seem to point, though not yet with any degree of certainty, in the same direction. This is a problem requiring the most searching investigation before any conclusions can be drawn.

Intemperate living in all its phases, and the strenuous life, are responsible for a large part of the modern abbreviation of life. Strenuous living is the result largely of a desire, in a short space of time, to accumulate enough to last throughout a long period of contemplated ease. The first part of this endeavor usually succeeds, but the last part of it fails of accomplishment in the greatest number of instances because of the lack of any life to live, after the strenuous period. Temperance in living must be preached.

The work of conserving and prolonging the life is now fairly launched, yet it still lacks the active support of the public at large; and it is here that physicians can and must help by their propaganda—for it seems that the physician's sphere is slowly narrowing itself down to the field of preventive medicine. The value of such work can be brought home to the public only by demonstrating the money value of each life saved to the community and thus to each individual. Prof. Irving Fisher, of Yale, estimates each life at a money value of $1,700. Civilization means materilization—but in a broader sense.

Little by little life has been prolonged, disease is being overcome, and life is worth living. We have now reached the average grand old age of fifty. Who knows what is to be the limit?—*Ed. N. Y. Med. Journal.*

